# Single hepatocytes show persistence and transcriptional inactivity of hepatitis B

**DOI:** 10.1172/jci.insight.140584

**Published:** 2020-10-02

**Authors:** Ashwin Balagopal, Tanner Grudda, Ruy M. Ribeiro, Yasmeen S. Saad, Hyon S. Hwang, Jeffrey Quinn, Michael Murphy, Kathleen Ward, Richard K. Sterling, Yang Zhang, Alan S. Perelson, Mark S. Sulkowski, William O. Osburn, Chloe L. Thio

**Affiliations:** 1 Department of Medicine, Johns Hopkins University School of Medicine, Baltimore, Maryland, USA.; 2 Theoretical Biology and Biophysics Group, Los Alamos National Laboratory, Los Alamos, New Mexico, USA.; 3 Faculdade de Medicina Universidade de Lisboa, Lisbon, Portugal.; 4 Division of Gastroenterology, Hepatology and Nutrition, Virginia Commonwealth University, Richmond, Virginia, USA.; 5 Department of Pathology, Johns Hopkins Hospital, Baltimore, Maryland, USA.

**Keywords:** Hepatology, Infectious disease, Hepatitis, Molecular biology, Transcription

## Abstract

There is no cure for the more than 270 million people chronically infected with HBV. Nucleos(t)ide analogs (NUCs), the mainstay of anti-HBV treatment, block HBV reverse transcription. NUCs do not eliminate the intranuclear covalently closed circular DNA (cccDNA), from which viral RNAs, including pregenomic RNA (pgRNA), are transcribed. A key gap in designing a cure is understanding how NUCs affect HBV replication and transcription because serum markers yield an incomplete view of intrahepatic HBV. We applied single-cell laser capture microdissection and droplet digital PCR to paired liver biopsies collected from 5 HBV/HIV-coinfected persons who took NUCs over 2–4 years. From biopsy 1 to 2, proportions of HBV-infected hepatocytes declined with adherence to NUC treatment (*P* < 0.05); we extrapolated that eradication of HBV will take over 10 decades with NUCs in these participants. In individual hepatocytes, pgRNA levels diminished 28- to 73-fold during NUC treatment, corresponding with decreased tissue HBV core antigen staining (*P* < 0.01). In 4 out of 5 participants, hepatocytes with cccDNA but undetectable pgRNA (transcriptionally inactive) were present, and these were enriched in 3 participants during NUC treatment. Further work to unravel mechanisms of cccDNA transcriptional inactivation may lead to therapies that can achieve this in all hepatocytes, resulting in a functional cure.

## Abstract 2

Single-cell laser capture microdissection integrated with droplet digital PCR was used to study hepatocytes from individuals with chronic hepatitis B.

## Introduction

Chronic HBV is the leading cause of hepatocellular carcinoma and end-stage liver disease globally. Although a vaccine exists for HBV, there is no cure for the more than 270 million people who are chronically infected (1). A major barrier to a cure is the covalently closed circular DNA (cccDNA) that resides and persists in the nucleus of every infected hepatocyte and that is the template for transcription of viral RNAs (2). Because current antiviral treatments can control HBV replication in the hepatocyte cytoplasm but are ineffective at eliminating cccDNA, a major research priority is to develop curative therapies for HBV that affect this cccDNA reservoir (3). However, despite advances in cell culture and animal models, we have few insights into human HBV and regulation of cccDNA transcription in the organ that it infects, the liver, especially in people receiving antiviral treatments.

Mature HBV virions contain relaxed circular DNA (rcDNA; Supplemental Figure 1; supplemental material available online with this article; https://doi.org/10.1172/jci.insight.140584DS1). Upon infecting the human hepatocyte, rcDNA uncoats and is converted into cccDNA by host DNA polymerases in the nucleus. Thereafter, a suite of short and long viral mRNAs is transcribed by host RNA polymerase II. These viral transcripts are exported into the cytoplasm and either translated into structural, regulatory, or replicative proteins, or packaged into new virions. Specifically, the long 3.5 kb RNA encodes for preC mRNA and pregenomic RNA (pgRNA); pgRNA is encapsidated as the template for the nascent virion or, as preC mRNA, is translated into the core and polymerase proteins that also compose the virion. The polymerase, a reverse transcriptase, converts the encapsidated pgRNA into rcDNA to restart the cycle of infection. The most commonly used current antivirals are nucleos(t)ide analogs (NUCs), often dually active against HIV and HBV, which inhibit the reverse transcriptase, thereby interrupting the conversion of pgRNA to rcDNA. However, since NUCs act in the cytoplasm, they are not known to have direct effects on cccDNA formation or on viral transcription. Intriguingly, recent reports have shown that plasma HBV RNA levels (largely pgRNA) decline with NUCs, albeit more slowly than plasma HBV DNA levels that largely comprise rcDNA (4–11). In our recent cross-sectional study of single hepatocyte analysis of HBV in situ, intracellular pgRNA levels were lowest in people who had prolonged NUC therapy (12).

Taken together, these observations raise the hypothesis that transcription of viral mRNA from cccDNA is downregulated during NUC treatment, but to test this directly requires advanced tools and liver specimens obtained from people taking NUCs. We developed single-cell laser capture microdissection (scLCM) to study viral hepatitis in the liver (13, 14) and recently adapted it to study HBV (12). We designed and validated specific primers and probes for different targets in the HBV genome and transcriptome, using droplet digital PCR (ddPCR) to quantify multiple viral targets with single-cell resolution. We applied the integrated platform to tissues collected from HBV/HIV-coinfected people who had core biopsies taken over the span of 2–4 years during NUC treatment through an ongoing natural history study. Our chief aims were to quantify residual HBV infection in situ during NUC treatment and to test directly whether NUC treatment resulted in decreased HBV transcription.

## Results

### Participant characteristics.

Participants in this study were all HIV coinfected because the parent Hepatitis B Research Network (HBRN) study focused on HBV/HIV coinfection (see Methods). From the tissues collected at Johns Hopkins University, 5 individuals with hepatitis B e antigen–positive (HBeAg-positive) chronic HBV, who had 2 biopsies, were selected for this study. They were selected based on a range of plasma HBV DNA levels (1.60–8.56 log_10_ IU/mL) at baseline (biopsy 1). Two individuals were not on HBV therapy at baseline but were on HIV therapy and had high plasma HBV DNA levels approximately 2 weeks before biopsy 1 (HB6 and HB2 had > 8 log_10_ IU/mL); 2 individuals were on dually active antiretroviral therapy (ART) containing NUCs with incomplete adherence and thus had intermediate HBV DNA levels (HB7 and HB3 had > 4 log_10_ IU/mL); and 1 individual was adherent to long-term NUC therapy with low levels of HBV DNA at biopsy 1 (HB4) (Table 1). Among them, the median (min, max) age at baseline was 47 (range 28–53) years, and all 5 were male and Black. All 5 had controlled HIV; specifically, plasma HIV RNA in HB6, HB7, and HB4 was undetectable, was between 241 and 358 cp/mL in HB2, and was 54 cp/mL in HB3. The median (min, max) baseline CD4^+^ T cell count was 390 (range 153–655) cells/μL. NUCs with antiviral activity against HBV between the 2 biopsies included tenofovir (as tenofovir disoproxil fumarate [TDF] or later as tenofovir alafenamide [TAF]) for 4 participants and entecavir (ETV) for HB2.

The time between biopsies ranged from 2.7 to 3.7 years. Because this was a registry and not a clinical trial, adherence to ART and NUCs was emphasized by clinicians in the study team and routinely by providers, but participants were not removed from the study for nonadherence. Thus, the participants had different HBV virological outcomes. HB2 and HB4 had HBV virological suppression at the time of biopsy 2, and HB6 had low levels of HBV DNA. HB7 and HB3 had residual viremia of more than 2 log_10_ IU/mL, implying relative nonadherence to NUCs compared with the other participants.

### scLCM and ddPCR for HBV nucleic acid targets.

We performed scLCM and ddPCR on the 2 biopsies that had been archived from each of these 5 participants (10 biopsies total). We isolated 100 to more than 200 hepatocytes per person per biopsy, for a total of 2080 hepatocytes. In each hepatocyte, we quantified 3 molecular viral targets (cccDNA, total HBV DNA, and pgRNA/preC mRNA; Supplemental Figure 2) and 1 host RNA (7SL) to assess for cell fragmentation, totaling more than 8000 separate measurements. Because ddPCR works on the principle of measuring individual PCR reactions in 20,000 to 40,000 droplets per sample, our results are derived from more than 180 million individual PCR reactions. The number of individual PCR reactions per HBV target per cell confers immense precision to our estimates of the abundance of each molecular target in each cell (see Methods and Supplemental Figure 3 for 95% confidence intervals around measurements).

### HBV-infected hepatocytes persist for years despite NUCs.

**Figure 1:**
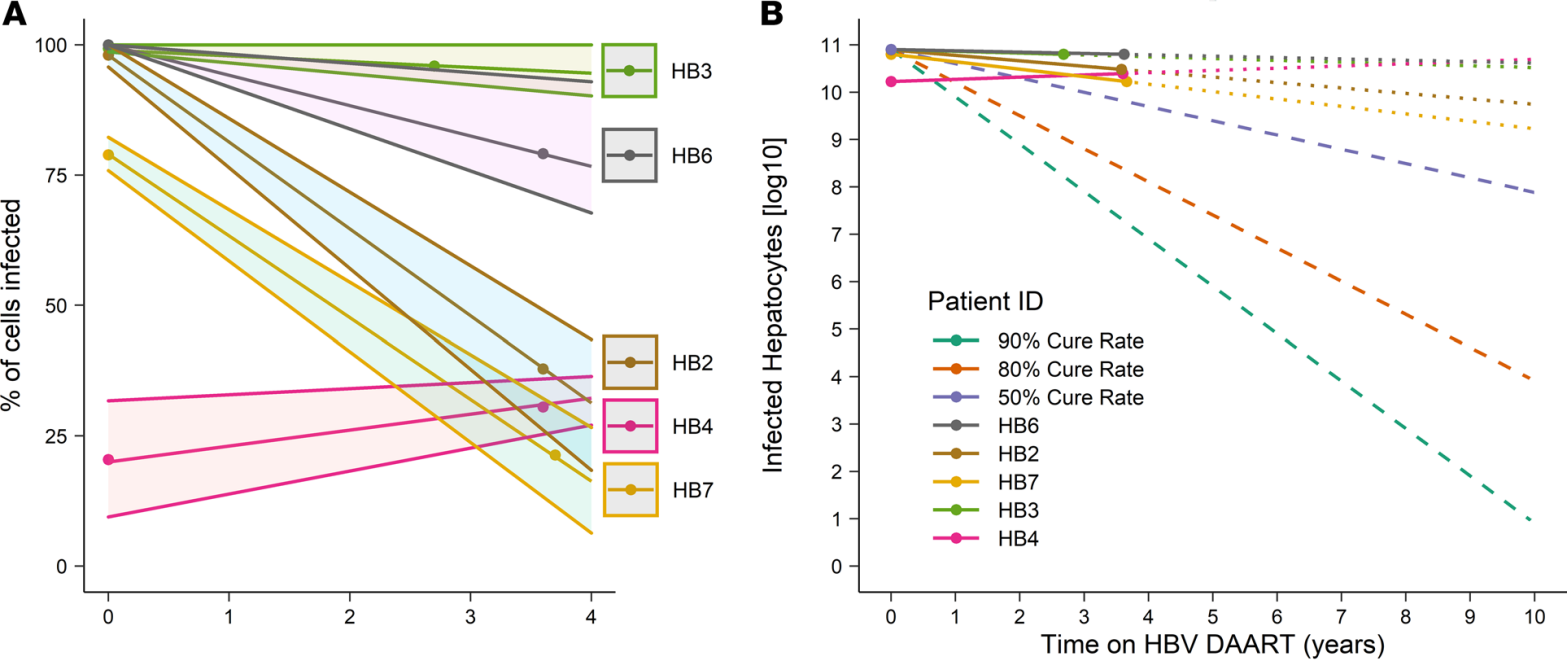
HBV eradication from the liver is slow. (**A**) The proportion of infected hepatocytes for each participant at each biopsy is shown. Points indicate when each biopsy was taken; the line indicates the inferred trajectories of the proportion infected, extrapolated from the proportions at each point; 95% confidence intervals are shown in the light shaded portion around each line. (**B**) The log_10_(number of infected hepatocytes) was estimated from the proportion of infected hepatocytes that was calculated at each liver biopsy in the 5 participants. The changes in log_10_(number of infected hepatocytes) for each participant were extrapolated to estimate when the liver could be considered eradicated of all infection. Extrapolated values are shown by dotted lines. For comparison, 90%, 80%, and 50% annual cure rates (i.e., the number of HBV-infected hepatocytes that are cleared each year) are shown as dashed lines. DAART, dually active antiretroviral therapy.

We first determined the proportion of hepatocytes infected at baseline and the change in this proportion between biopsies. We defined a hepatocyte as infected if any of the 3 HBV-specific targets were detectable in the cell, with pgRNA detection being more sensitive for infection than the DNA markers (Supplemental Table 1). In biopsy 1, the proportion of HBV-infected hepatocytes was more than 95% in HB6, HB2, and HB3; 79% in HB7; and a smaller but still significant proportion, 20%, in HB4, the individual on long-term NUCs with low/undetectable plasma HBV DNA before biopsy 1 (Table 2 and Figure 1). In the 2 participants who initiated NUCs near the time of biopsy 1, plasma HBV DNA levels dramatically declined between biopsies: from more than 8 log_10_ IU/mL at baseline to 1.63 log_10_ IU/mL and undetectable in HB6 and HB2, respectively. Correspondingly, the proportion of infected hepatocytes in HB6 declined from 100% to 79% and in HB2 from 98% to 38%. Plasma HBV DNA for HB7 declined between biopsies but was still detectable at biopsy 2 (2.89 log_10_ IU/mL), and the proportion of infected hepatocytes decreased from 79% to 21%. HB3 had a plasma HBV DNA level of 4.55 log_10_ IU/mL at biopsy 1 and 2.93 log_10_ IU/mL at biopsy 2, confirming poor adherence to NUCs, and the proportion of infected hepatocytes did not change appreciably, from 99% to 96%. In contrast, HB4 had undetectable HBV viremia at and between both biopsies, and the proportion of infected hepatocytes notably did not decrease but remained relatively stable at 20% to 31% over 3.6 years.

Next, we estimated the rates of decline in the proportion of infected hepatocytes: using mixed-effects modeling, we found a significant, but very slow, decline in the number of infected hepatocytes over time (*P* < 0.05; Supplemental Table 2). We extrapolated from these values to the time it would take to eradicate HBV from the liver in these individuals, assuming constant adherence, the absence of drug resistance, and a continuous log-linear decline in the number of infected hepatocytes at the same rates as observed between biopsies (Figure 1). We also estimated the time to eradication of HBV for a model therapy that would lead to loss of 90%, 80%, or 50% of infected hepatocytes annually. We found that in the 5 people who we studied, continued antiviral therapy with NUCs would be unlikely to result in a cure because the median decline in the number of infected cells was 10-fold every 20 years, implying it would take more than 10 decades to attain a cure. Even with optimistic modeling of a 90% annual loss of infected hepatocytes, the time to eradication from the liver would be nearly 10 years.

### NUCs are associated with decreased transcription of cccDNA.

**Figure 2:**
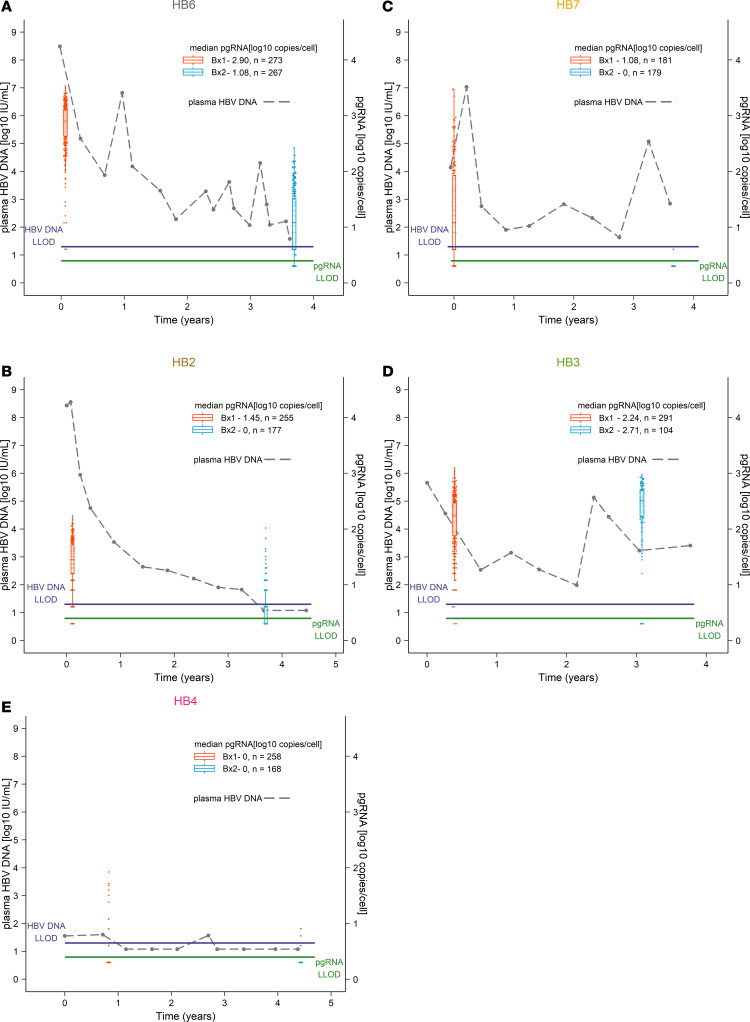
Virologic control of HBV with NUCs is accompanied by reduced transcription of pgRNA. Shown in dashed lines and on the left axes are the plasma HBV DNA levels spanning the interval between the 2 core liver biopsies for (**A**) HB6, (**B**) HB2, (**C**) HB7, (**D**) HB3, and (**E**) HB4. Shown in box plots and on the right axes are the aggregated single-cell pgRNA levels at the first (red box-and-whisker and individual points) and second (blue box-and-whisker and individual points) biopsies. For each point in the box plots, 20,000–40,000 individual PCRs were performed. The lower limits of detections for plasma HBV DNA (shown in purple) and intracellular pgRNA (shown in green) are also shown. Median log_10_ pgRNA copies/cell are shown in the legend for each participant.

As expected, NUC therapy decreased the proportion of hepatocytes with total intracellular HBV DNA levels (*P* = 0.01; Supplemental Table 2) and levels of total HBV DNA levels per hepatocyte in people who also had observable decreases in plasma HBV DNA levels (*P* = 0.02; Supplemental Table 3 and Supplemental Figure 4). Next, to test our hypothesis that NUCs decrease cccDNA transcription, changes in pgRNA levels were determined in hundreds of hepatocytes between biopsies. Surprisingly, in individual hepatocytes, levels of intracellular pgRNA, which have not been described to be directly affected by NUCs (Supplemental Figure 1), decreased in the group as a whole, but not in all participants (*P* = 0.02; Table 2 and Supplemental Table 3), and more strikingly in those (HB6 and HB2) who had declines in plasma and intracellular HBV DNA and were adherent to NUCs (*P* = 0.0001). Specifically, pgRNA levels in HB6 decreased from a median (min, max) of 2.91 (0.60–3.53) log_10_ cp/cell to 1.04 (undetectable–2.42) log_10_ cp/cell (Table 2 and Figure 2A) and in HB2 decreased from a median (min, max) of 1.45 (undetectable–2.24) log_10_ cp/cell to below the limit of detection (undetectable–2.02) log_10_ cp/cell (Table 2 and Figure 2B). The proportion of hepatocytes that contained pgRNA also declined for HB6 and HB2 (*P* < 0.01; Supplemental Table 2). For HB7, who had incomplete adherence but still had a plasma HBV DNA decline from 4.15 to 2.89 log IU/m, the median (min, max) pgRNA declined from 1.08 (undetectable–3.47) log_10_ cp/cell to undetectable (undetectable–0.60) log_10_ cp/cell (Table 2 and Figure 2C). Two participants had relatively stable pgRNA levels: HB4, who had low or undetectable plasma HBV DNA throughout, and HB3, who had incomplete adherence and whose plasma HBV DNA did not decline substantially (Table 2 and Figure 2, D and E).

**Figure 3:**
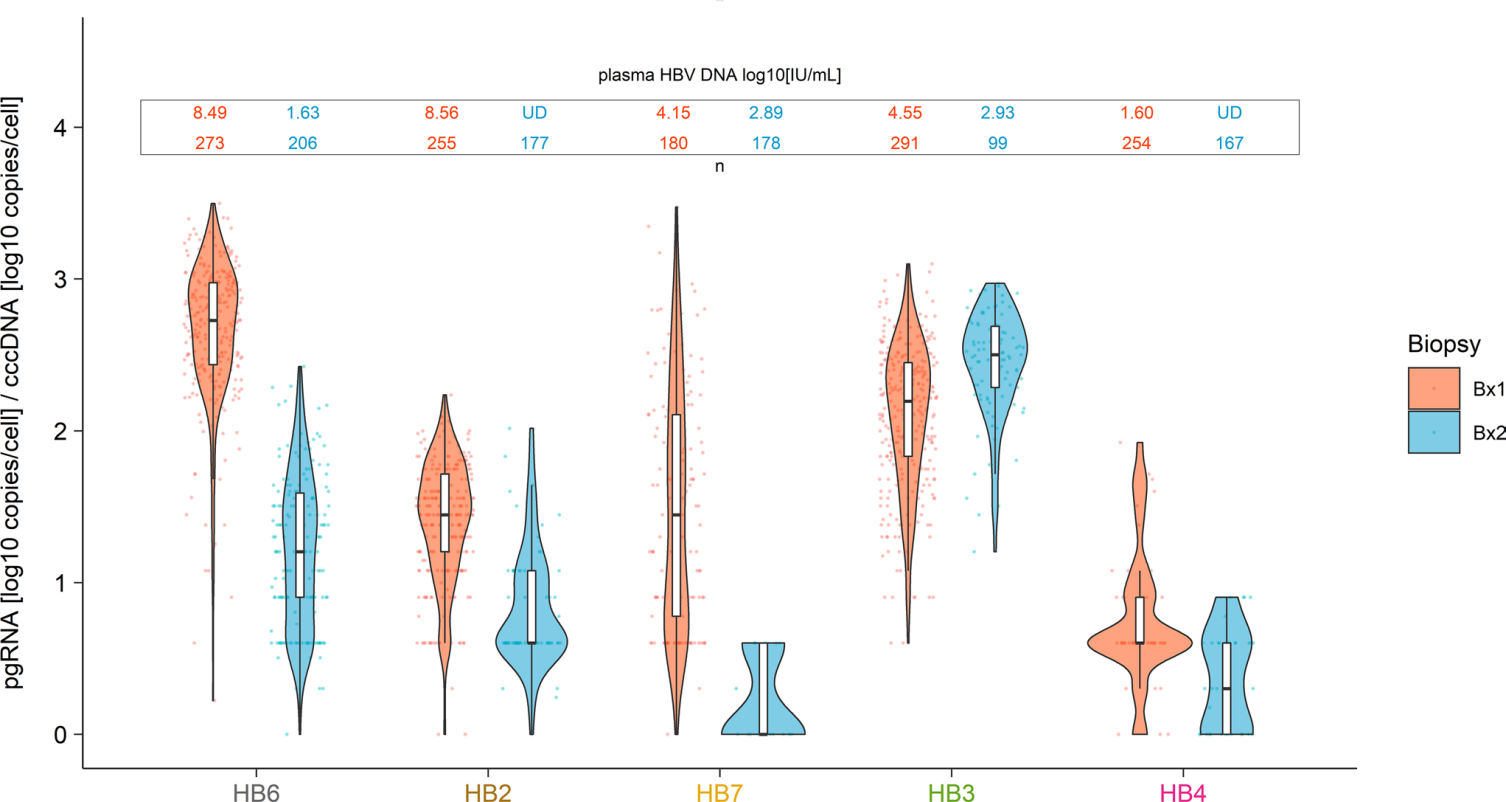
The transcriptional index is reduced when plasma HBV DNA levels decrease. The transcriptional index was calculated for each cell by dividing the number of pgRNA copies by the number of cccDNA copies in the same cell (*x* axis). Violin plots (showing medians, IQRs, distributions, and individual points) show the transcriptional index for every cell, stratified by participant, in the first (salmon) and second (light blue) biopsies. Shown above each pair of violin plots are the contemporaneous plasma HBV DNA levels (above) and the number of examined hepatocytes (below). Values are log_10_ transformed; therefore, hepatocytes that did not contain pgRNA or cccDNA are not shown.

Although these results were consistent with a change in viral transcriptional activity during NUC therapy, the alternate possibility is that NUCs led to diminished reinfection and replenishment of cccDNA, and thus the amount of pgRNA decreased proportionally. However, there were no significant changes in the proportion of hepatocytes containing cccDNA (Supplemental Table 2) or in the number of cccDNA molecules per hepatocyte between the paired biopsies (Table 2 and Supplemental Table 3), although the range of cccDNA did appear to change for HB6 (Supplemental Figure 5). To confirm these observations, we calculated a transcriptional index for each cell as the number of pgRNA copies per cccDNA in a given cell (Table 2 and Figure 3). We found that the median transcriptional index in biopsy 1 for participant HB6 was 533 (min 1, max 3145) and at biopsy 2 was 16 (min 0, max 264). Similar declines in the transcriptional index were observed in HB2 and HB7 with observable declines in plasma HBV DNA, strongly supporting that there was a decline in HBV transcription with NUC therapy in those who also had suppressed plasma HBV DNA levels (Table 2 and Figure 3). The wide range of transcriptional indexes within an individual also supported variability in transcription between hepatocytes with the extreme of no transcription (transcriptional index of 0). Indeed, for 4 individuals (HB6, HB2, HB7, HB4), we found evidence of infected cells with detectable cccDNA but undetectable pgRNA (cccDNA^+^/pgRNA^–^). These cells, which were either transcriptionally silent or transcriptionally inactive, were a larger proportion of all infected cells in people with demonstrated NUC adherence at biopsy 2 (Table 2). Surprisingly, 1 of those individuals, HB4, who had suppressed HBV viremia at both biopsies, had an increase in the proportion of cells that were transcriptionally inactive from 1.2% to 7.8%. Importantly, as a proportion of only infected cells, these transcriptionally inactive cells increased in HB4 from 5.8% to 25.5%, suggesting that long-term NUC adherence increases the proportion of infected cells that are transcriptionally inactive.

### Declines in hepatitis B core antigen production correlate with decreased pgRNA transcription during NUC treatment.

**Figure 4:**
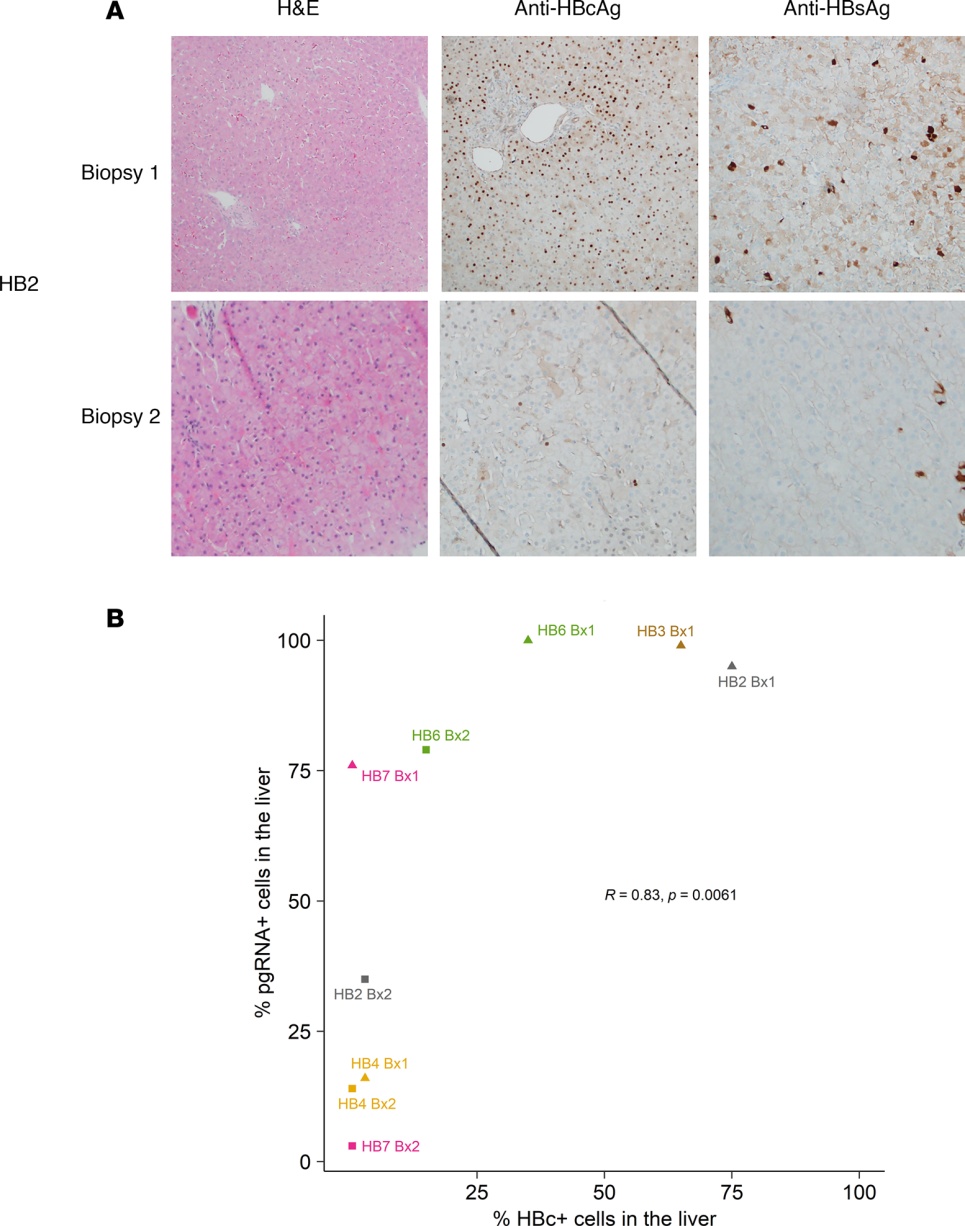
HBV core protein expression is reduced during NUC treatment and corresponds with the intracellular transcriptional index. (**A**) IHC was used to stain for HBcAg and HBsAg in first and second biopsies for all 5 participants. Shown are representative micrographs for HB2, alongside hematoxylin and eosin staining. (**B**) The amount of staining for HBcAg was quantified in each biopsy by a hepatopathologist who was blinded to participant identity (Supplemental Table 4). Shown is the correlation between the midpoint percentage of HBcAg-stained cells and the percentage of hepatocytes that contained detectable pgRNA, indexed by participants and time point. Data for HB3 biopsy 2 are not shown because less than 30% of tissue was available for staining; this slide was not further analyzed. Spearman’s correlation coefficients and associated *P* values are shown in **B**.

Since pgRNA also encodes for the HBV core protein via preC mRNA, we hypothesized that pgRNA levels would correlate with the amount of hepatitis B core antigen (HBcAg) in liver tissue. Using IHC for staining HBcAg and hepatitis B surface antigen (HBsAg) as a positive control, the proportion of hepatocytes with HBcAg staining correlated with the proportion of cells that had detectable pgRNA (*r* = 0.83; *P* = 0.0061; Supplemental Table 4 and Figure 4, A and B). We noted that pgRNA detection by ddPCR was more sensitive than HBcAg detection, as there was a range of detection of pgRNA when HBcAg stained less than 5% of hepatocytes. Taken together, our scLCM/ddPCR results and IHC strongly indicated that HBV underwent transcriptional regulation of its cccDNA such that pgRNA (which encodes for precore and core antigens and is the genomic template for new virions) diminished with NUCs.

## Discussion

In this intensive longitudinal study of intrahepatic single-cell HBV replication, using tissues collected from HBV/HIV-coinfected people taking ART containing NUC therapy, we performed scLCM on more than 2000 hepatocytes and collected ddPCR data from more than 180 million individual PCR reactions. We synthesized these data to report 2 observations. First, we found that even with adherence to a potent antiviral regimen currently available, including tenofovir, the loss of HBV-infected hepatocytes was negligible. In addition, we found that NUC-mediated suppression of plasma HBV DNA levels was also associated with a decrease in intracellular pgRNA levels without a proportionate decrease in cccDNA, strongly suggesting viral transcriptional regulation during NUC treatment, a likely unique finding in vivo. These results underscore the main challenges in curing chronic HBV with NUCs but also offer clues as to how HBV may persist despite NUC therapy.

Single-cell quantification of the burden of HBV infection in the liver has been challenging and, to our knowledge, this is the first investigation of single-cell HBV infection in longitudinal liver biopsies. However, several studies have explored the rate at which HBV can be cleared from the liver during NUC therapy. Using bulk liver tissue, the decline in cccDNA in monoinfected persons was estimated at 0.80 log_10_ copies/cell after 48 weeks of NUC therapy (15). Similar results have been reported in HBV/HIV-coinfected people during tenofovir therapy (16). Animal models have shown variable rates of cccDNA decline with NUC therapy. A model of duck HBV infection demonstrated rapid cccDNA decline with NUC therapy that ultimately reached a plateau (17). Similarly, cccDNA decline in woodchuck HBV with NUC therapy has been described as quite slow compared with intrahepatic total HBV DNA loss (18). In vitro loss of cccDNA is similarly slow (19). Our results are not directly comparable to these prior reports because we quantified infected cells. By focusing on the infected cell, we can estimate the time to loss of all infected cells, the sine qua non of an eradicative cure. In the case of a functional cure, the infected hepatocyte is still the relevant unit since it is likely that the biology of the hepatocyte governs the transcriptional activity of the cccDNA reservoir within.

Several recent reports describing the kinetics of HBV RNA in blood during NUC treatment support our findings of NUCs affecting transcriptional regulation. It is first important to note that plasma HBV RNA is thought to be encapsidated intracellular HBV RNA (20). Plasma HBV RNA has been increasingly used as a novel biomarker of chronic hepatitis B (CHB). Intriguingly, numerous investigations consistently find that plasma HBV RNA levels decrease during NUC treatment, albeit at intermediate rates between rapidly declining plasma HBV DNA levels and the very slow rates of HBsAg decline. Persons with CHB who received NUCs for 4–64 months experienced declines in plasma HBV RNA levels between 3 and 6 months after initiation of treatment (4). Plasma HBV RNA levels are correlated with both plasma HBV DNA levels and with HBsAg levels and appear to predict HBeAg and HBsAg loss in some patients. There is a close relationship between plasma HBV RNA levels and intrahepatic HBV RNA levels, which adds support to our findings (21, 22).

Plasma HBV RNA levels have been shown to predict response to pegylated interferon (8, 11, 23) and to NUC interruption (6, 7, 9, 10): persons with suppressed plasma HBV DNA and RNA levels are less likely to develop HBV reactivation after NUC interruption than persons with suppressed HBV DNA levels but detectable HBV RNA levels. One potential explanation for this finding is that persons with suppressed HBV RNA levels might have infected cells that are transcriptionally inactive, and thus less likely to reactivate. Notably, one group reported that detectable blood HBV RNA levels were found in people who later experienced HBV reactivation, suggesting that silent cccDNA can be stimulated to actively transcribe, thus beginning anew a cycle of viral replication and, consequently, hepatitis (i.e., transaminase elevations). It is tantalizing to consider transcriptional regulation of cccDNA as the linchpin of a functional cure. Indeed, siRNAs to the HBV S gene have recently resulted in prolonged suppression of blood HBsAg levels (24). It is possible, therefore, that the transcriptionally inactive or silent cell that we have identified in the liver is both the goal of a functional cure strategy, and potentially the reservoir from which HBV reactivates. Thus, understanding how HBV transcription is regulated, and how it can be durably suppressed, may be a key step in developing an HBV cure.

HBV transcriptional regulation has been extensively reviewed elsewhere (25). There are numerous transcription factors and 2 viral enhancers that could be modified during NUC treatment. Briefly, we conjecture that multiple aspects of the biology of the infected cell should be explored in vivo, including innate immune signaling, microRNA suppression, positive and/or negative feedback from viral gene products, and epigenetic modifications. We further conjecture that although all types of transcriptional regulation may culminate in epigenetic silencing, proximate molecular causes, or “switches,” should be sought to understand how to design therapies that silence HBV effectively.

Our study had several limitations that warrant mention. Our sample size is clearly limited and only includes Black males. This was largely because of the demographics of the enrollment area. The limited sample size, however, is offset by intensive study of each individual at 2 time points, providing a more comprehensive analysis of intrahepatic HBV in these participants, and by the consistency in the findings. Moreover, our findings are clearly consistent with larger studies characterizing plasma HBV RNA kinetics during NUC therapy. Ours is the first study, to our knowledge, to quantify HBV replication at the single-cell level during a longitudinal perturbation. In that regard, we have demonstrated the feasibility of similarly designed translational studies to examine HBV biology during a therapeutic intervention. It is also worth remarking that we only studied tissues from HBV/HIV-coinfected people. Although studies of coinfection are important for this neglected special population, one question that arises from this approach is whether our findings are generalizable to HBV-monoinfected people. Most participants had preserved CD4^+^ T cell counts, supporting that our results are generalizable. However, while we acknowledge that our study should be repeated in HBV-monoinfected people, we also note that the importance of our findings, their consistency, and their explanatory value to the condition of HBV monoinfection outweigh the sample selection. It is also important to note that among our assays, the pgRNA assay appeared to be most sensitive. Thus, it is possible that cccDNA rates declined more robustly than observed.

In closing, we report here that integrated scLCM and ddPCR can be applied to HBV in situ to reveal a dynamic viral landscape (viroscape) during antiviral treatment. We estimated the number of infected cells and the change in the number of infected cells with NUC therapy, quantifying the gap between current strategies and ideal eradicative cure scenarios. We also demonstrated that HBV transcription of pgRNA declined nearly 100-fold during NUC therapy. We conclude that durable transcriptional silencing may be an effective strategy to achieve a functional cure, although there remains the possibility of HBV reactivation. Further work is needed to understand HBV transcriptional regulation during treatment as a potential route to a cure.

## Methods

### Participant selection.

Liver tissues were obtained from 5 people with chronic HBV (defined as HBsAg positive) and HIV-1 coinfection (defined as HIV-1 antibody positive), through the HIV-HBV Cohort Ancillary Study of the HBRN (R01 DK094818; ClinicalTrials.gov NCT01924455). Through the HBRN registry, paired core liver biopsies were collected by ultrasound-guided biopsy within an interval of 2–4 years. At the time of procurement, all tissues were placed in neutral cutting media and stored in liquid nitrogen until use.

### Plasma HBV DNA levels.

Plasma HBV DNA levels were determined by the Johns Hopkins Clinical Laboratory.

### scLCM, DNA/RNA extraction, and reverse transcription.

scLCM was performed on more than 2000 hepatocytes from all subjects, separately isolating each hepatocyte into individual microfuge tubes containing proprietary lysis buffer (ZR-Duet DNA/RNA MiniPrep kit, Zymo Research), as we have done previously (12). DNA and RNA from each hepatocyte were extracted separately according to the manufacturer’s protocol, except using Zymo-Spin IC columns and eluting in 22 μL of nuclease-free water. RNA extracts were treated with DNase I in-column during the extraction, and SuperScript IV First-Strand Synthesis System (Thermo Fisher Scientific, 18091050) was used to synthesize complementary DNA (cDNA) according to the manufacturer’s protocol.

### Molecular assay characteristics for HBV DNA replicative forms.

We adapted primers and probes for total HBV DNA, cccDNA, and pgRNA to a ddPCR format that we have previously validated for low-abundance targets (Supplemental Figure 2) (15, 26, 27). We used enzymatic treatments with exonuclease I/III treatments (exo I/III; New England Biolabs, M0293 and M0206) to diminish the false detection of rcDNA as cccDNA by cleaving DNA that is not fully circular (28). We have previously shown that this method clears all of the noncircular HBV DNA (rcDNA and integrated DNA) before analysis (12).

### ddPCR.

DNA extract from each cell was divided equally in 2 portions: each one-half portion of DNA extract (11 μL) from each cell was used to quantitate either total HBV DNA or cccDNA. One-quarter portion of cDNA (11 μL) was used to quantitate pgRNA. DNA and cDNA were solubilized in water with Bio-Rad ddPCR Supermix for Probes (No dUTP) (1863023/1863024/1863025), primers, and fluorescent probes, and loaded into droplet generation cartridges. Subsequently, single samples were partitioned with oil to form thousands of droplets through microfluidics channels. Droplet-partitioned samples were then transferred to 96-well plates, and PCR was performed as we have described previously (12). Plates were then read by the QX200 Droplet Digital PCR System (Bio-Rad), an endpoint fluorescence detection reader, according to the manufacturer’s protocol. As a quality control measure, 2 cells were excluded from further analysis because fewer than 10,000 droplets were formed from their cellular extracts. The concentration of target in a sample was calculated using Poisson statistics as a function of the number of droplets that were positive for the PCR product and the number of droplets that were negative for the product. Since the sensitivity of ddPCR is partially constrained by the total number of droplets in which PCR was performed, we performed the assay twice for total HBV DNA and cccDNA to lower the limit of detection. Consequently, the lower limits of detection for total HBV DNA and cccDNA, when calculated, were 2 copies per cell and for pgRNA were 4 copies/cell, similar to what was reported by Laras et al. (27). As a means of calibration, ddPCR was run with 4 polyethylene naphthalate membrane controls (DNA negative) per 100 hepatocytes, assigning any cell with this value or less as having no HBV. Using the raw numbers of positive and negative droplets for each cell, we calculated 95% confidence intervals, conservatively estimating the error for each quantity (Supplemental Figure 2).

### Real-time reverse transcription quantitative PCR.

Real-time reverse transcription quantitative PCR was used to detect 7SL, an abundant cytoplasmic RNA that is used to normalize the amount of captured cytoplasm for each hepatocyte. 7SL was quantified in cDNA using PrimeTime Gene Expression Master Mix (Integrated DNA Technologies, 1055770) and compared with a cloned 7SL standard as we have done previously (13). Captures with less than 1 standard deviation below a standard negative control measurement were excluded from the final analysis to avoid false quantification of cell fragments. Negative controls for cellular material included empty portions of the same slides that did not contain liver tissue.

### IHC.

Glass slides were stained at our institution with IHC stains for hepatitis B core and surface antigens. Slides were baked at 95°C for 36 minutes and loaded onto a BenchMark ULTRA slide stainer (Ventana Medical Systems). Briefly, stains were deparaffinized and heat-induced epitope retrieval was achieved with Tris-based ULTRA CC1 solution (Ventana Medical Systems).

For HBsAg staining, a polyclonal rabbit antibody against HBcAg (Abcam, ab115992) was used at a dilution of 1:300 and incubated for 32 minutes. Detection was achieved using an ultraView Universal DAB detection kit (Ventana Medical Systems), and slides were counterstained, dehydrated, and mounted. Tissue with known hepatitis B core antigen expression was used as a positive control.

For HBsAg staining, a predilute monoclonal mouse antibody against HBsAg (Cell Marque, A10F1) was used and incubated for 28 minutes. Detection was achieved using an iVIEW DAB detection kit (Ventana Medical Systems), and slides were counterstained, dehydrated, and mounted. Tissue with known hepatitis B surface antigen expression was used as a positive control.

### Statistics.

Because ddPCR relies on thousands of negative droplets in a measurement to determine concentration, 95% confidence intervals were calculated when the standard error of the mean was unusable because of extreme outlier values. We employed 3 calculations: A = 2*r* + 3.84; B = 1.96 × [3.84 + (4*r*(*n* – *r*)/*n*)]^½^; C = 2(*n* + 3.84), where *n* is the total number of droplets and *r* is the number of positive droplets in a single ddPCR reaction. The 95% lower and upper confidence intervals were then calculated as (A – B)/C and (A + B)/C, respectively.

We analyzed the decay of infected cells with different genomic material components using mixed-effects models. In this approach, we used all the data together to fit a regression model, where we assumed that participants were a sample from a given population and entered the model as a grouping (or random) effect. Time between biopsies was the independent variable, such that we calculated the decay of hepatocytes containing a given viral target (total HBV DNA, cccDNA, or pgRNA) per year. We also used mixed-effects models to analyze the decay of different viral targets per infected cell, using the median levels of cp/cell. Here, we calculated the decay of each viral target per year.

Transcriptional indices were compared between biopsies 1 and 2 using a pairwise Wilcoxon’s rank sum test in R (“pairwise.wilcox.test”). *P* values less than 0.05 were deemed significant. Spearman’s correlation coefficients were calculated in R (“stat.cor”; method = Spearman) comparing the percentage of hepatocytes stained positive for HBcAg to the percentage of hepatocytes positive for pgRNA extrapolated from grid measurements. Any *r* values approaching 1 with a *P* value less than 0.05 were deemed significant.

### Study approval.

The use of tissues from humans for the present study was reviewed and approved by the Office of Human Subjects Research Institutional Review Board, IRB-3, Baltimore, Maryland, USA. All participants gave written informed consent for use of their tissues for research purposes through the HBRN.

## Author contributions

AB conceived of the study, provided funding, performed analyses, and drafted the manuscript. TG performed experiments, performed analyses, and verified data integrity. RMR performed analyses and edited the manuscript. YSS performed experiments. HSH performed experiments, performed analyses, and verified data integrity. JQ performed quality control and verified data integrity. MM edited the manuscript. KW performed recruitment of all participants and managed the study database. RKS was responsible for collecting samples and edited the manuscript. YZ performed IHC and analysis and edited the manuscript. ASP performed analyses and edited the manuscript. MSS conceived of the study, was responsible for collecting samples, and edited the manuscript. WO edited the manuscript. CLT conceived of the study, provided funding, performed analyses, and drafted and edited the manuscript. All authors approved the final version of the manuscript.

## Supplementary Material

Supplemental data

## Figures and Tables

**Table 1:**
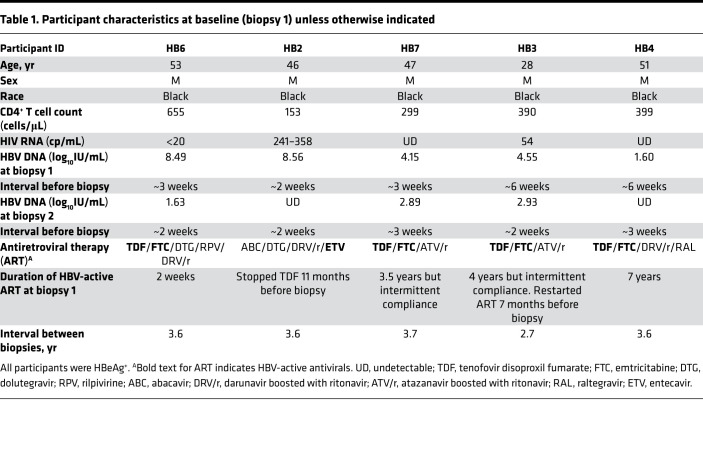
Participant characteristics at baseline (biopsy 1) unless otherwise indicated

**Table 2:**
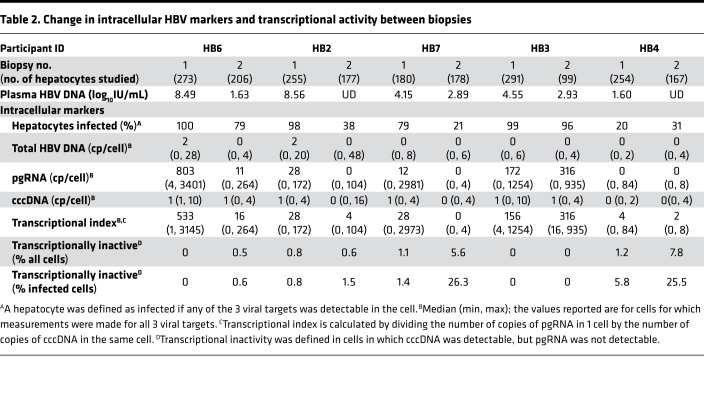
Change in intracellular HBV markers and transcriptional activity between biopsies

## Version 1 10/02/2020

Electronic publication
