# Supplementary material for: Single hepatocytes show persistence and transcriptional inactivity of hepatitis B
Source: JCI Insight. 2020 Oct 2;5(19):e140584. doi: 10.1172/jci.insight.140584 (PMC7566712; doi:10.1172/jci.insight.140584)
Supplement: Supplemental data [file jciinsight-5-140584-s110.pdf]

Supplemental materials.

Supplemental Tables.

Table S1. Proportion of hepatocytes positive for each molecular target.

Participant ID	Biopsy	%pgRNA+ cells (95% CI)	%Total HBV DNA+ cells (95% CI)	%cccDNA+ cells (95% CI)
**HB6**	1	**100** (100-100)	**62.8** (38.5-87.1)	**32.3** (7.7-57.0)
	2	**80.2** (70.1-90.3)	**6.6** (2.3-11.0)	**8.7** (0-20.3)
**HB2**	1	**76.3** (72.2-80.4)	**12.8** (11.2-14.4)	**8.3** (7.6-9.1)
	2	**3.3** (0-9.7)	**13.4** (11.9-14.8)	**6.1** (1.0-11.2)
**HB7**	1	**16.3** (7.5-25.1)	**4.4** (0.9-7.8)	**2.4** (0-7.1)
	2	**14.2** (8.2-20.2)	**9.7** (4.4-14.9)	**10.4** (0-21.5)
**HB3**	1	**95.0** (93.7-96.3)	**59.4** (37.6-81.2)	**6.9** (0-14.0)
	2	**34.7** (19.7-49.8)	**4.6** (2.0-7.1)	**3.9** (3.1-4.7)
**HB4**	1	**99.3** (98.7-100)	**24.2** (16.3-32.1)	**9.9** (0-22.3)
	2	**97.6** (93.0-100)	**12.6** (0.1-25.2)	**1.2** (0-3.5)

Shown are the proportions of hepatocytes for each participant at each biopsy that had detectable targets for pgRNA, total HBV DNA, or cccDNA. In parentheses are the 95% confidence intervals.

Table S2. Rates of decline of infected hepatocytes with each molecular target.

Molecular Target	Rate of decline (all participants) $\log_{10}$ [molecular target positive hepatocytes/year][a]	p-value[b] (all participants)	Rate of decline (HB6 and HB2 only[c]) $\log_{10}$ [molecular target positive hepatocytes/year][a]	p-value[b] (HB6 and HB2 only[c])
**Total HBV DNA**	-0.13	0.01	-0.28	<0.0005
**cccDNA**	NC	NA	NC	NA
**pgRNA**	NC	NA	-0.07	<0.01
**Any target**	-0.05	0.02	-0.07	<0.01

[a]Analyses of the decay of infected cells with different genomic material components using mixed-effects models. In this approach, all data were used together to fit a regression model where we assume participants are a sample from a given population and enter the model as grouping (or random) effect. Time between biopsies is the independent variable, such that we are calculating the decay of cells containing each viral target.
[b]p-value indicates whether decay rate is different than zero. $P<0.05$ is considered significant.
[c] HB6 and HB2 were analyzed separately as these individuals were untreated at the time of biopsy 1 and had low or undetectable plasma HBV DNA levels at the time of biopsy 2.
NC – not calculated since rate of decline is not significant; NA - not applicable

Table S3. Rates of decline of HBV molecular target per hepatocyte.

Molecular Target	Rate of decline (all participants) log_{10} [cp/hepatocyte/year][a]	p-value[b] (all participants)	Rate of decline (HB6 and HB2 only[c]) log_{10} [cp/hepatocyte/year][a]	p-value[b] (HB6 and HB2 only[c])
**Total HBV DNA**	-0.03	0.02	-0.07	0.01
**cccDNA**	NC	NA	NC	NA
**pgRNA**	-0.35	0.02	-0.34	0.0001

[a]Analyses of the decay of different genomic material components per infected cell using mixed-effects models on the median levels cp/cell. In this approach, we use all data together to fit a regression model where we assume patients are a sample from a given population and enter the model as grouping (or random) effect. Time between biopsies is the independent variable, such that we are calculating the decay of genomic material per year.
[b]p-value indicates whether decay rate is different than zero. $P<0.05$ is considered significant.
[c]HB6 and HB2 were analyzed separately as these individuals were untreated at the time of biopsy 1 and had low or undetectable plasma HBV DNA levels at the time of biopsy 2.
NC – not calculated since rate of decline is not significant; NA - not applicable

Table S4. Histologic staging and immunohistochemistry for viral proteins.

	Biopsy 1				Biopsy 2			
**Participant ID**	**Grade[a]**	**Stage[a]**	**Core Ag[b]**	**Surface Ag[c]**	**Grade[a]**	**Stage[a]**	**Core Ag[b]**	**Surface Ag[c]**
**HB6**	A0	F0	30-40%	1-5% **[C]**<br>>95% **[M]**	A1	F0	10-20%	5-10% **[C]**<br>70-80% **[M]**
**HB2**	A1	F1	70-80%	20-30% **[C]**<br>90-95% **[M]**	A0	F0	1-5%	1-5% **[C]**<br>70-80% **[M]**
**HB7**	A1	F2	<1%	10-20% **[C]**	A0	F2	<1%	10-20% **[C]**
**HB3**	A0	F0	60-70%	5-10% **[C]**<br>>95% **[M]**	A1	F0	<1%[d]	5-10% **[C]**<br>>95% **[M]**
**HB4**	A1	F0	1-5%	<1% **[C]**	A1	F0	<1%	none

[a]Grading and Staging of liver disease was performed using the METAVIR scoring system.
[b]All HBcAg staining was noted to be nuclear.
[c]HBsAg staining was noted to be either cytoplasmic **[C]** or membranous **[M]**.
[d]<30% of tissue was available for staining; this slide was not further analyzed.

Supplemental Figure 1.

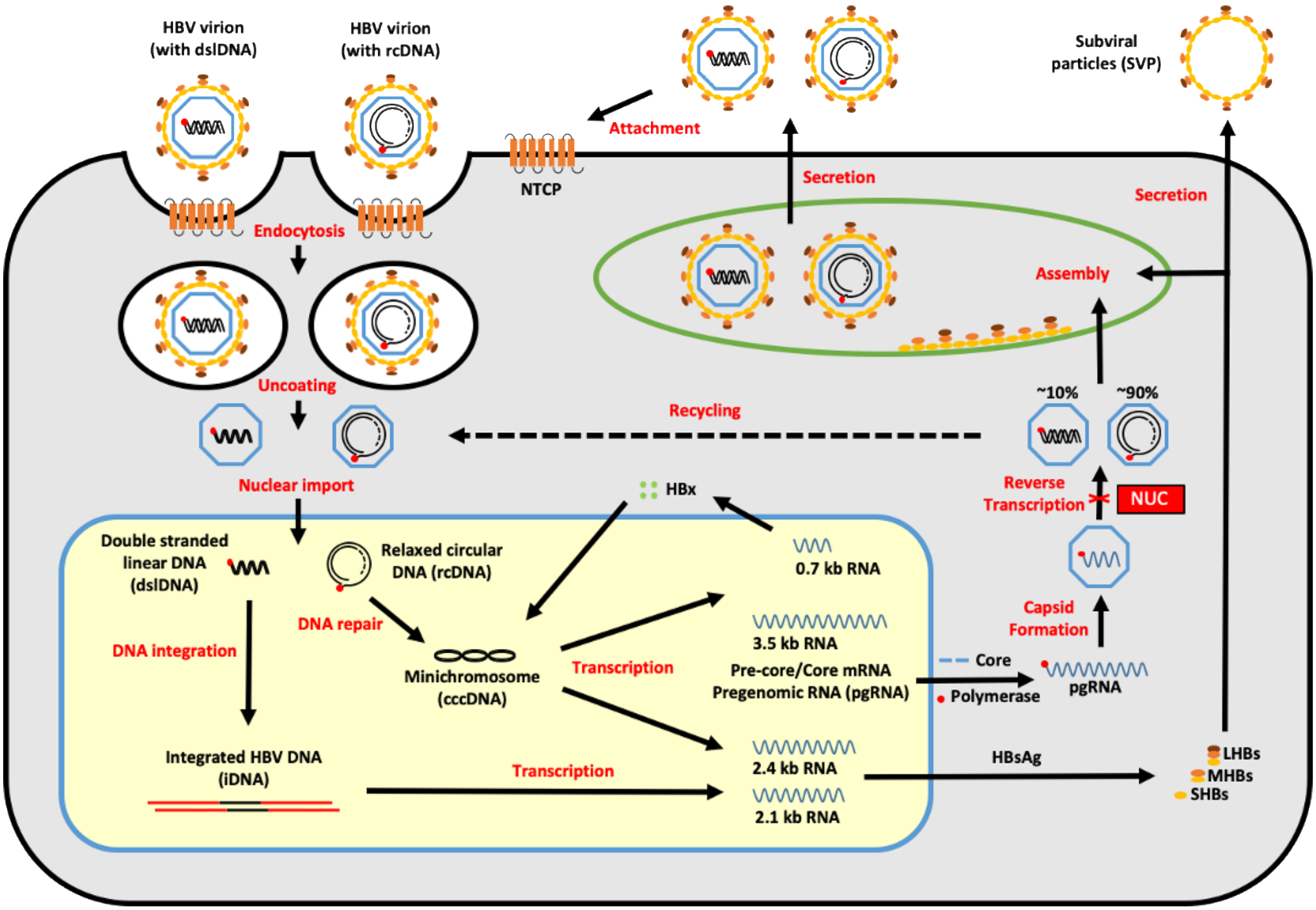

SFig. 1. The HBV lifecycle, viral transcription, and the action of NUC. The illustration summarizes the key steps in the HBV lifecycle, including entry of the virion that contains rcDNA, uncoating of the virion, the production of cccDNA, viral transcription and translation, formation of the capsid, reverse transcription, viral assembly and encapsidation, and viral egress. Also shown are integration of HBV into the human genome and the production of HBsAg, either from transcripts deriving from cccDNA or integrated HBV DNA. Noted in a red text box is the putative action of NUCs in interrupting reverse transcription, thus diminishing rcDNA production. There is no known role of NUCs on viral transcription.

Supplemental Figure 2.

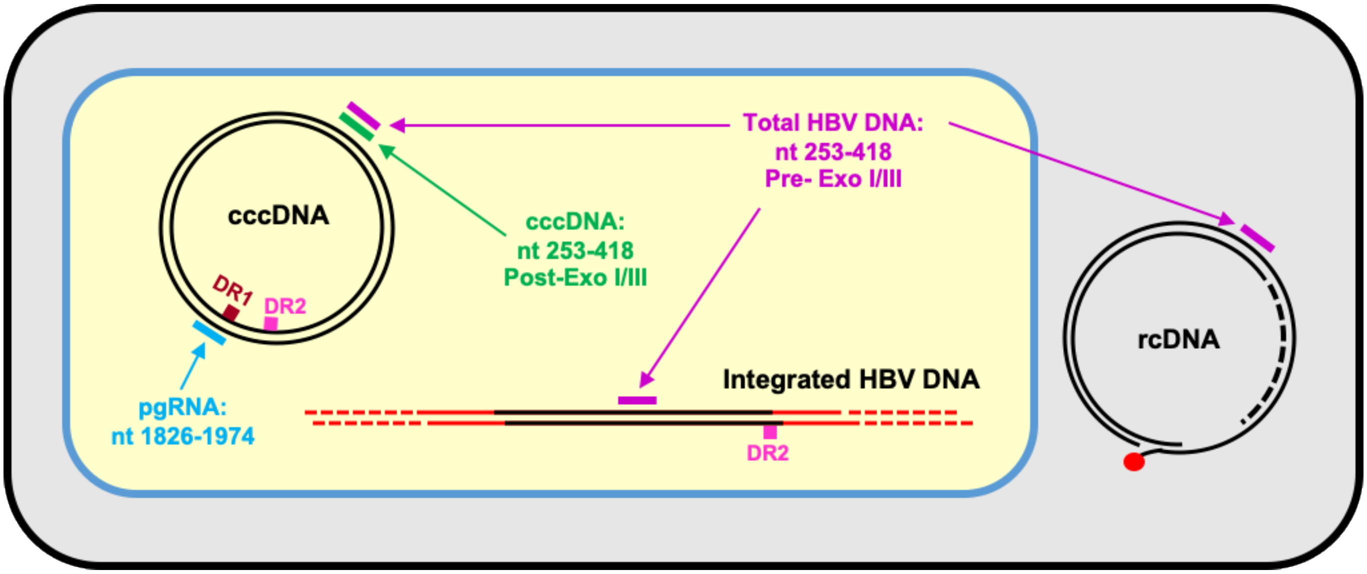

SFig. 2. Molecular map show the sites which were targeted by ddPCR assays. Each target is delineated by a separate color with genomic positions indexed to cccDNA.

Supplemental Figure 3.

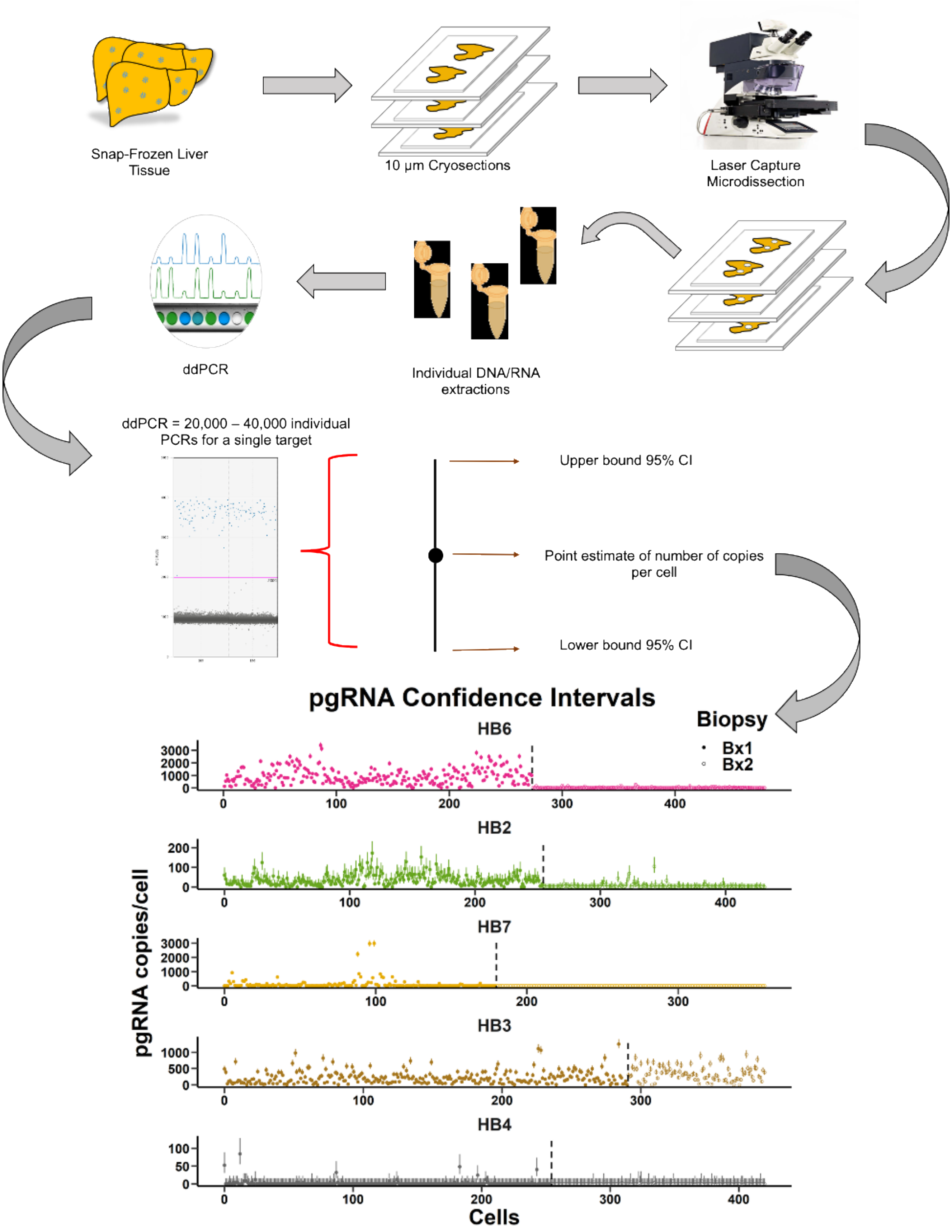

SFig. 3. Schema of data acquisition. Archived snap frozen liver tissues are cryosectioned onto PEN membrane slides. Single-cell laser capture microdissection (scLCM) is performed to individually isolate hepatocytes into separate cryofuge tubes. Each cell has its RNA and DNA extracted separately. In the example shown, RNA is extracted and droplet digital PCR (ddPCR) is performed for pgRNA. Droplet positivity and total number of droplets are used to generate confidence intervals for the number of molecular targets in a cell. Shown as an example are pgRNA copies per cell: 20-40,000 droplets are analyzed by the ddPCR reader for fluorescence. Positive droplets are separated by their fluorescence intensity from negative droplets using a set threshold (horizontal pink line). Poisson statistics are used to calculate the point estimate of the number of copies of pgRNA per cell, as well as an upper and lower bound of the 95% confidence intervals (CI) for the estimate. Shown are each of the analyzed cells (indexed in the x-axes) in biopsy 1 (Bx1; closed symbols) and 2 (Bx2; open symbols), separated by vertical hashed lines for each participant, with their respective 95% confidence intervals.

Supplemental Figure 4.

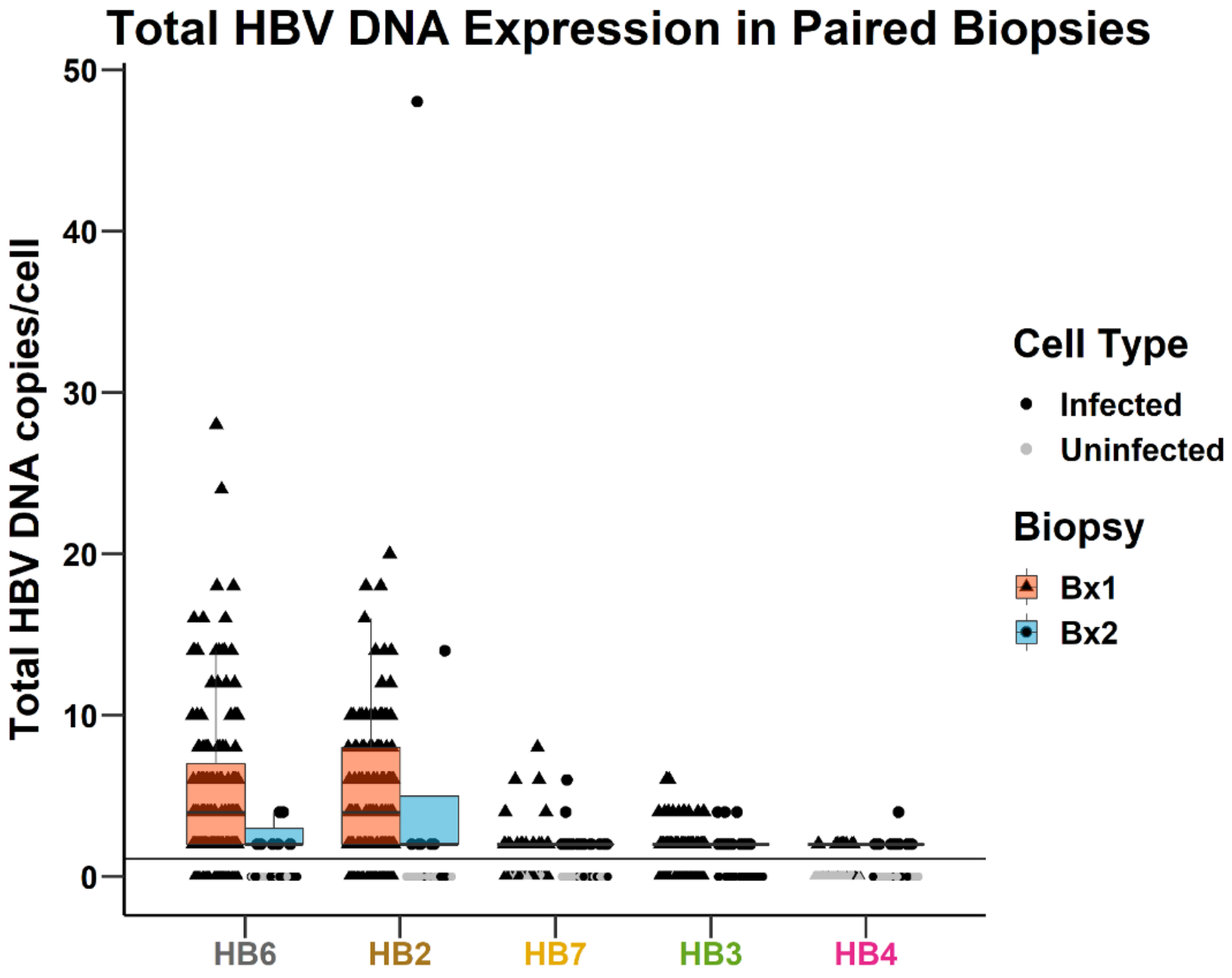

SFig. 4. NUC changes the abundance of intracellular total HBV DNA in infected cells. Shown are box-plots of single-cell total HBV DNA levels for each person at biopsy 1 (salmon boxes; triangles; Bx1) and biopsy 2 (light blue boxes; circles; Bx2)(each point shows a single cell).

The limit of detection is demarcated by the solid black horizontal line. Infected cells (black) and uninfected cells (grey) are distinguished in the diagram below the solid horizontal line.

Supplemental Figure 5

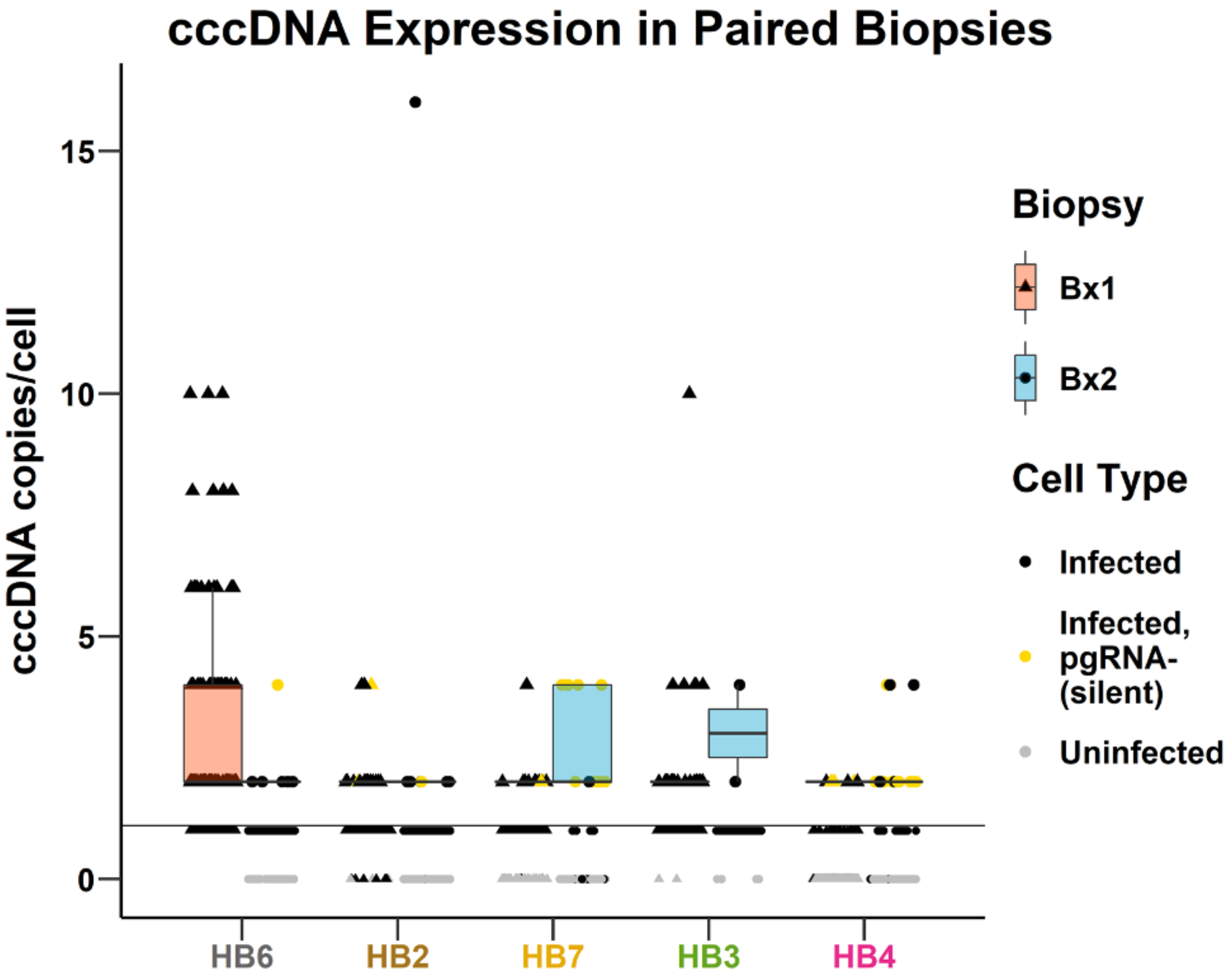

SFig. 5. NUC do not change the abundance of intracellular cccDNA in infected cells. Shown are box-plots of single-cell cccDNA levels each person at biopsy 1 (salmon boxes; triangles; Bx1) and biopsy 2 (light blue boxes; circles; Bx2)(each point shows a single cell). Infected cells are shown as either transcriptionally active (black), transcriptionally inactive (yellow), or uninfected (grey).

Cells that had cccDNA levels below the limit of detection (solid black horizontal line) but that were determined to be infected (because of the detection of a different viral marker) were assigned a conservative value of 1 copy/infected cell. Cells that had cccDNA levels below the limit of detection are denoted by gray symbols (uninfected – no detectable nucleic acid targets) or black symbols (infected – either detectable pgRNA, total HBV DNA, or both). The number of infected cells with undetectable cccDNA numbered only 13 across all biopsies.
